# Regional Collaborative Forecast of Cargo Throughput in China's Circum-Bohai-Sea Region Based on LSTM Model

**DOI:** 10.1155/2022/5044926

**Published:** 2022-07-07

**Authors:** Junfei Cui, Bingchun Liu, Yan Xu, Xiaoling Guo

**Affiliations:** ^1^Guorong Securities Co., Ltd., Tianjin Huangpu South Road the Sales Department, Tianjin 300201, China; ^2^School of Management, Tianjin University of Technology, Tianjin 300384, China

## Abstract

Any developed port plays a dominant role both in domestic and international trade reflecting economic prosperity of the port and nearby regions in terms of its cargo throughput and port construction. An attempt is made in this study to use long-and short-term memory (LSTM) artificial neural network method to construct the port cargo throughput prediction model. Three ports namely, Tianjin Port, Dalian Port, and Tangshan Port from China's Bohai Rim region are selected as research objects. The historical cargo throughput of each port for nearly ten years was used as the input index data for joint prediction. The cargo throughput of Bohai Port provides another way to improve the accuracy of port cargo throughput prediction. The prediction results show that the LSTM model can effectively predict the port cargo throughput; the cargo throughput forecasts between the three Bohai Rim ports have both an interactive relationship and differences.

## 1. Introduction

The port is a vital transit point for domestic and foreign trade. A well planned port logistics shortens the transportation time and distance of various trades, boosts trade exchanges, and plays an important role in improving national economy. Developed countries in the world generally have their own coastlines and well-functioning ports, and the large ports become gateways for foreign trade. On the 5,800-kilometer coastline of China's Bohai Sea, there are more than 60 large and small ports competing among themselves namely, competition between different port groups; competition between different ports of the same port group; competition between different enterprises in the same port. How a port is in a dominant position in the process of port integration is particularly important for accurate port planning and construction. The port construction involves a wide range of issues, which are related to the construction of railways, highways, pipeline layouts, and agricultural and industrial production development of a city or region. The construction of the port requires a long gestation period and huge infrastructure investment. The problem of underconstruction or overconstruction results either in congestion and delay or waste of space, time, and decline in expected income, respectively.

Port cargo throughput is an important indicator of the port's operational capacity [1]. There are many factors affecting port cargo throughput, such as politics, economy, transportation facilities, cultural differences, natural environment, and climate change. Accurate prediction of port cargo throughput can provide a more reliable reference for decision makers to develop port investment plans, rational development, rationale, and protection of national port resources. At present, the methods for predicting port throughput mainly include traditional econometric models, artificial intelligence models, and hybrid models. Econometric models are mostly used for time series analysis. By extracting feature data and meaningful statistical data, future values are predicted based on historical values. There are mainly exponential smoothing models [2], autoregressive (AR) [3] and moving average models [4], autoregressive moving average model (ARMA) [5], autoregressive comprehensive moving average model (ARIMA) [6], and the broader autoregressive slightly integrated moving average model (ARFIMA) [7]. The artificial intelligence model uses computer algorithms to decompose the raw data time series into different neural network input variables to predict future values. The most widely used method in the prediction field is the artificial neural network (ANN) [8] and support vector machine (SVM) [9]. LSTM neural network is a long-term and short-term memory network. It is used for large-scale parallel processing, distributed storage, and processing, and is also suitable for inaccurate information problems that need to consider many influencing factors and conditions, and can effectively solve nonlinear prediction problems [10]. At present, the deep learning algorithm is rarely used in port cargo throughput forecasting. Port throughput is affected by many factors, showing nonlinearity and volatility, competing with the world economy and surrounding ports, since the environment is closely related. Using the LSTM algorithm to predict the monthly cargo throughput of the Bohai Rim port can improve the accuracy of predicting port cargo throughput.

In the past, the forecasting of port cargo throughput was usually based on the target port's throughput indicator, the target port hinterland economic indicator, and the neighboring port's throughput indicator as input indicators to predict a target port. This study believes that port cargo throughput may be affected by the fluctuation of cargo throughput in the adjacent port, in addition to the operations of its own port and the economic development of the port hinterland. To this end, the present study selected three ports around the Bohai Sea namely, Tianjin Port, Dalian Port, and Tangshan Port as the research objects, and established a cooperative prediction model of port cargo throughput based on the LSTM deep learning method. Firstly, by considering the correlation of cargo throughput between ports in the same region, 12 forecasting schemes were designed to predict port cargo throughput. Secondly, most of the raw data was used to train the LSTM model to get the best parameters. In addition, a small portion of the raw data is entered into the optimal LSTM model to obtain predictions and errors. Finally, the analysis of experimental results was used to verify the interactions between the three ports.

Port economy and development-related issues have been a popular topic of research among scholars, most of whom only focus on the ports themselves, while demand forecasting and related research on port-related indicators are less. In this paper, the research on the current situation and the degree of future synergistic development of port clusters is innovative in terms of application practice. In this paper, LSTM is used as an experimental model to treat the Bohai Sea port cluster as a whole system for prediction experiments. The main contributions and innovations are as follows: (1) This paper applies the LSTM model to port-related research by constructing an LSTM model to predict the nonlinear time-series data of port-related characteristic indicators. (2) By dividing the ports into subgroups by administrative divisions, the paper provides new ideas for the prediction of port cargo throughput by cross-predicting the historical data of different ports. (3) The LSTM model prediction results are compared with those of other models, and it is determined that the LSTM model is applicable to port research.

## 2. Literature Review

### 2.1. Demand Forecasting

Demand forecasting is already a mature research topic in many areas such as port throughput, air passengers, energy consumption, and air quality. Several prediction methods and models have been proposed in literature which can be divided into three categories, namely econometric models, artificial intelligence models, and hybrid models.

Econometrics models are applied to time series analysis by extracting meaningful data features and statistics to observe future values based on past observations. When predicting univariate time series data, simple methods such as naive model and exponential smoothing model can be used to represent the random process of time series. The naive model uses the latest value as a prediction for the next period, while the exponential smoothing model uses a recursive calculation method that updates each new input observation as it is predicted. Merkuryeva et al. [11] used SMA models, multiple linear regression, and genetic programming regression to study product demand predictions in the drug supply chain and discussed their practical implications. The above method can provide better prediction results under linear assumptions but cannot accurately predict demand with nonlinear characteristics. Candelieri et al. [12] proposed a parallel global optimization model to optimize the hyperparameters of support vector machines and predicted water resources. Suh and Ryerson [13] used this information to develop very efficient scheduling systems and AI-enabled demand forecasting models.

The artificial intelligence model is a kind of intelligent activity that recognizes, classifies, memorizes, associates, predicts, etc., through computer simulation of human thinking process. It has self-learning, self-organization, and self-adaptive ability, and can fit arbitrary functions, especially nonlinear functions. In general, econometric models and time series are influenced by the stability of historical data and economic structures, while artificial intelligence focuses on the size and quality of training data. Artificial neural network (ANN), minimum models such as two-square support vector regression (LSSVR), and support vector machine (SVM) provide a more effective solution for nonlinear prediction problems. Takashi Tanizaki et al. [14] proposed a machine learning-based catering demand forecasting method and established a demand forecasting model including store location, events, and weather. Plakndaras et al. [15] predicted the transportation needs of aviation, roads, and trains in the US domestic market based on econometric models and machine learning models. The results show that support vector regression (SVR) is better than the least absolute shrinkage and selection operator. Law et al. [16] used a large number of search intensity indicators of tourism demand and studied the monthly passenger arrivals in Macao through deep learning methods. The proposed method is more accurate than the artificial neural network model and support vector regression (SVR).

Recurrent neural network (RNN) is a deep network structure that utilizes sequence information (Cho et al., 2014). RNN selectively processes data elements by passing information across time steps, a feature that is important for requirement forecasting. Embedded structures in time series data convey useful contextual information in requirement forecasting. Matyjaszek et al. [17] studied the performance of traditional time series models, autoregressive integrated moving average (ARIMA) model, generalized regression neural networks (GRNN), multilayer feedforward networks (MLFNs), and robust models in predicting coking coal prices. The prediction results show that the prediction accuracy of GRNN model is higher than that of other models. RNN can maintain storage units in the hidden layer to receive information from previous state to current state, which is suitable for prediction problems based on time series data [18]. Convolutional neural networks (CNNs) can be combined with air quality layer characteristics to construct prediction models that improve prediction accuracy over traditional multiple linear regression models [19]. Zhao used 1-D and 2-D CNNs with LSTM network for speech emotion recognition [20].

LSTM is an extension of RNN. As an effective and scalable model, LSTM has been widely used in speech recognition, handwriting recognition, image analysis [21], document abstract [22], and music construction. Learning problems are related to sequence data such as modulo and control robots. Yildirim et al. [23] used the LSTM network for data analysis of electrocardiogram (ECG) signals to aid in the automatic detection of arrhythmia signals. Makarenkov et al. [24] demonstrated and evaluated the effectiveness of LSTM in terms of lexical substitution (LS) and grammatical error correction (GEU). Majd and Safabakhsh [25] designed a deep network for human motion recognition using the LSTM unit extended version C2LSTM and confirmed the effectiveness of C2LSTM in capturing motion, spatial characteristics, and time dependence by UCF101 and HMDB51. Kim [26] showed that convolutional neural networks (CNNs) and LSTM have good performance in speech emotion recognition, especially the two-dimensional CNN LSTM network, which is significantly better than the traditional dynamic Bayesian network DBN. Whereas, CNN LSTM is also commonly used in demand forecasting, and Yu et al. [22] proposed an improved long-short-time memory-enhanced forgotten gate network model (LSTM-EFG) for wind power forecasting. Compared with other predictive models, the prediction accuracy is improved by 18.3%. Yang et al. [27] proposed the LSTM method to overcome the problems of different lengths of traffic flow data, irregular sampling, and missing data. The multiscale time smoothing method was used to infer the missing data, and the prediction error was obtained. Experiments show that LSTM has higher accuracy in traffic flow prediction than other methods. In addition, there are some research methods based on attention mechanism in time series prediction tasks that can also achieve high prediction results [28, 29]. This technology can also effectively support efficient and intelligent forecasting of household electricity demand [30].

At present, the deep learning algorithm is less applied in port cargo throughput forecasting. The port throughput is affected by many factors, showing nonlinearity and large volatility, which is closely related to the world economy and the surrounding port competition environment. Using the LSTM algorithm to predict the monthly cargo throughput of the Bohai Rim port can improve the accuracy of predicting port cargo throughput.

### 2.2. Collaborative Forecasting

Collaborative prediction is a common method used to improve prediction accuracy. Cheikhrouhou et al. [31] proposed a mathematical demand forecasting judgment collaborative method based on the identification and classification of specific events, and the fuzzy inference system to evaluate the factors corresponding to specific events to ensure the consistency of the prediction results. Empirically, this method improves the accuracy of demand forecasting. Chen [32] proposed a collaborative artificial intelligence method to accurately predict the cost of semiconductors and established a cooperation mechanism. The effectiveness of the method was demonstrated by an example. About collaborative artificial intelligence method, experts predict the unit cost through FBPN based from his own point of view. Each expert communicates prediction results to other experts under the synergy mechanism. After receiving the information, the experts can change their settings according to the cooperation mechanism. Gao [33] continuously monitors outage risks based on coordinated forecasts of advance supply signals such as financial health and operational viability. The results show that collaborative prediction has high value for high margin products, medium fixed costs, and low demand fluctuations. The users are discussed. Wang and Toly Chen [34] improved the fuzzy cooperative intelligence (FCI) method, which can directly consider the original value of the yield. The results show that the DS-FCI method optimizes the prediction accuracy. Thomson et al. [35] incorporated a measure of coherence into an analytic evaluation framework using UK retail price index inflation forecasts for the period 1998–2014. Chen [36] constructed a collaborative fuzzy-neural agent network in order to predict global CO_2_ concentration, and the effectiveness of the method was verified by actual data.

### 2.3. Port Throughput Forecasting

The predecessors have two main aspects of port research, one is the study of the relationship between economic activities and port operations; on the other hand, the demand forecast of port cargo throughput. Kim et al. [26] consider the impact of exchange rate changes, global economic activity, and the volatility of the baltic dry index (BDI) on the cargo throughput of Korean ports, and study its influence relationship based on the error correction model. The results show that BDI volatility has a negative impact on freight volume, while nominal exchange rates and increased global economic activity have a positive impact on the freight volume. Rashed et al. [37] based on an empirical analysis of the annual total container throughput of major ports in the Hamburg-Le Havre area and some economic indicators show that there is a long-term relationship between the trade index of the EU19 and container throughput relationship. Gosasang et al. [38] used multilayer perceptron (MLP) and linear regression to predict the future container throughput of Bangkok Port. The factors affecting the cargo throughput of Bangkok Port (exchange rate, national economy, social development, energy consumption, etc.) are input into MLP and linear regression prediction models for cargo throughput forecasting. Xie et al. [39] predict container throughput by selecting a separate prediction model for each component based on data characteristic analysis (DCA).

Cargo throughput is one of the important factors affecting the economic development of the port. Accurate forecasting of cargo throughput not only improves the efficiency of port operations but also provides a reference for planning port development. Based on the analysis of the location advantage of Ningbo–Zhoushan Port, Li Zengwei and Ye Jun used the three-exponential smoothing method to analyze the port cargo throughput in the next five years. Patil GR and Sahu P K used multiple regression models and time series models to predict shipments from Mumbai, India, between 2014-15 and 2017-18, with the former being more accurate than the latter. Seabrooke et al. used regression analysis to predict the growth and development of freight in Hong Kong ports. C Zhang and L Huang used the combined model of grey forecasting model and logistics growth curve model for port cargo throughput forecasting and applied the model to an actual port in China, verifying the validity of the joint model, and the model has no geographical restrictions on external conditions such as culture. Mo et al. [40] constructed a hybrid prediction model based on GMDH (HFMG) based on container throughput data of Xiamen Port and Shanghai Port. The results show that the prediction performance of the HFMG model is superior to the SARIMA model and mixed prediction models such as SARIMA-svr, SARIMA-gp, and SARIMA-bp. To improve the accuracy of container throughput prediction, Niu et al. [41] established a hybrid decomposition integration model vmd - arima - hgw - svr (VMD), which has good predictive ability for container throughput data. Li et al. [42] proposed a quadratic decomposition learning method. The extreme value learning machine (KELM) combines to achieve monthly container throughput prediction. The empirical results show that the SD learning method is an effective model for predicting nonlinear nonstationary container throughput. Du et al. [43] studied the use of effective decomposition techniques and established a new hybrid learning model for container throughput prediction.

In the past, the forecasting of port cargo throughput was usually based on the throughput index of the target port and the economic indicators of the target port hinterland as input indicators to predict the throughput of the target port. At present, there are few studies on collaborative prediction between multiple ports. This study believes that port cargo throughput may be affected by the fluctuation of cargo throughput in the adjacent ports, in addition to the operations of its own port and the economic development of the port hinterland.

## 3. Methodology and Model

### 3.1. LSTM

**Figure 1:**
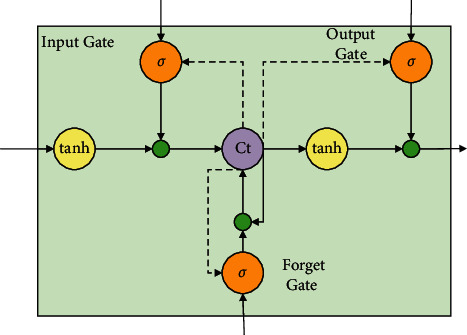
Memory unit structure.

The long short-term memory (LSTM) is a special recurrent neural network first proposed by Sepp Hochreiter and Jurgen Schmidhuber in 1997. Compared with the traditional cyclic neural network RNN, it is also based on time series data, through the cyclical connection between neurons, to find the relationship between time series data and modeling time series data. Different from RNN, LSTM solves the problem which the gradient of RNN often faces in the actual application process. The LSTM neural network model is similar to RNN with a hidden layer, but the nodes in each common hidden layer are replaced by storage units. Its core idea is a special neuron structure - “memory unit,” which can maintain its state over time. The nonlinear gating unit can adjust the flow of information into and out of the unit, as shown in Figure 1.

Suppose that at time *t*, the input, output, and state of a memory unit module are *x*_*t*_, *h*_*t*_, and *C*_*t*_, Then, the input gate, the forgetting gate, the input conversion, the unit state update, and the hidden layer output formula of the memory unit module are respectively shown in equations (1–6).

(1) $$\begin{array}{c} i_{t}=\sigma \left(W_{xi}x_{t}+W_{hi}h_{t-1}+b_{i}\right), \end{array}$$

(2) $$\begin{array}{c} f_{t}=\sigma \left(W_{xf}x_{t}+W_{hf}h_{t-1}+b_{f}\right), \end{array}$$

(3) $$\begin{array}{c} O_{t}=\sigma \left(W_{xo}x_{t}+W_{\text{h}o}h_{t-1}+b_{o}\right), \end{array}$$

(4) $$\begin{array}{c} C_{t}^{\prime }=\tanh\left(W_{xC}x_{t}+W_{hC}h_{t-1}+b_{C}\right), \end{array}$$

(5) $$\begin{array}{c} C_{t}=f_{t}y⊗C_{t-1}+i_{t}⊗{\tilde{C}}_{t}, \end{array}$$

(6) $$\begin{array}{c} h_{t}=o_{t}⊗\;\tanh\left(C_{t}\right), \end{array}$$

where *σ* is the sigmoid function; tanh is the hyperbolic tangent function; *i*_*t*_, *f*_*t*_, *o*_*t*_, and *C*_*t*_′ are the input gate, forgetting gate, output gate. and input conversion input to the unit, respectively; *W*_*xi*_, *W*_*xf*_, *W*_*xo*_, *W*_*xC*_ and *W*_*hi*_, *W*_*hf*_, *W*_*ho*_, *W*_*hC*_ are, respectively, the weight matrix of input gate, forgetting gate, output gate, and input transformation corresponding to *x*_*t*_ and *h*_*t*−1_. *b*_*i*_, *b*_*f*_, *b*_*o*_, and *b*_*C*_ are the offset vectors of input gate, forgetting gate, output gate, and input transformation, respectively.

According to *x*_*t*_ and *h*_*t*−1_, input transformation generates new information ${\tilde{C}}_{t}$ through function tanh; the input gate reads *x*_*t*_ and *h*_*t*−1_, and outputs values from 0 to 1 through the sigmoid function (0 indicates that no information is allowed to pass through, 1 indicates that all information is allowed to pass through); multiply this by the amount of information ${\tilde{C}}_{t}$ to determine how much new data information can be fed into the memory unit. At the same time, the forgetting gate reads the values of *x*_*t*_ and *h*_*t*−1_, outputs the values from 0 to 1 through sigmoid function (0 means complete abandonment, 1 means all reservation), and multiplicate them with the state of the moment on the memory unit to determine the retention and abandonment of the original information in the memory unit. The new information is then added to the retained original information to update the state of the current memory unit to *C*_*t*−1_. Finally, the output gate reads the values of *x*_*t*_ and *h*_*t*−1_, operates through the sigmoid function, and multiplicates the memory unit state 7 processed by the tanh function to determine the output information of the memory unit state.

**Figure 2:**
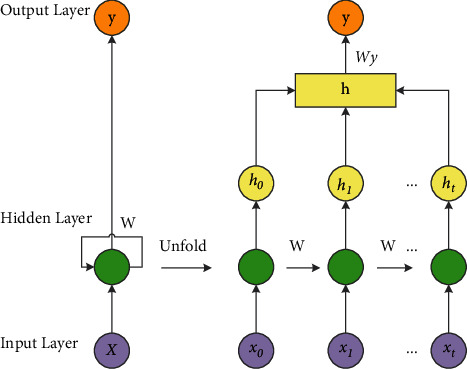
Single-layer LSTM network expansion structure.

The LSTM neuron adds a unit state to the traditional cyclic neurons to preserve long-term information. Therefore, the LSTM network has the same structure as the traditional cyclic neural network and consists of an input layer, a hidden layer, and an output layer. In the hidden layer structure, the connection from the hidden layer at the previous moment to the hidden layer at the next moment is added, and the unfolded structure of the single-layer LSTM network according to time is shown in Figure 2.

### 3.2. Design of Experiment and Model

**Figure 3:**
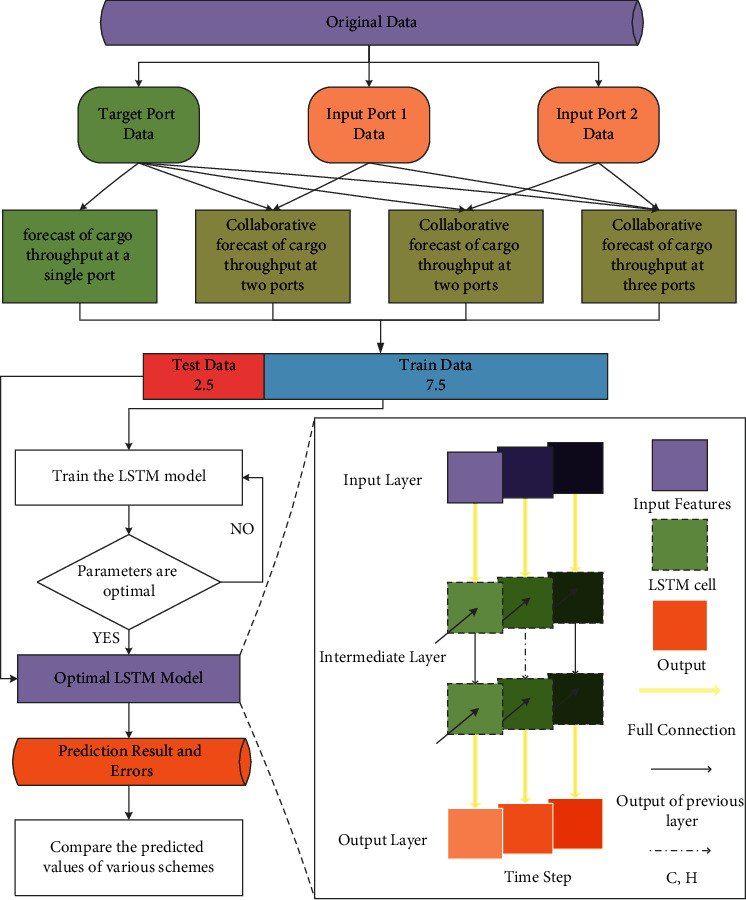
Experimental design and model.

For the coordinated forecast of the cargo throughput of the three ports around the port of Tianjin Port, Dalian Port, and Tangshan Port, the experiment was completed in five steps. The experimental design and the best LSTM model are shown in Figure 3.

In the first step, the forecasting experiments are divided into three types: target port forecasting, two-dimensional port cooperative forecasting, and three-dimensional port cooperative forecasting. There are 12 forecasting schemes. The target port forecasting scheme separately uses the historical monthly cargo throughput data of the target port as the input variable of the forecasting model and uses the next month's cargo throughput of the target port as the output variable, and then applies the LSTM model for prediction. The two-dimensional port collaborative prediction scheme uses the historical monthly port cargo throughput of the target port and the other two ports (input port 1 and input port 2) as the input variables of the prediction model, and takes the cargo throughput of the target port for the next month as the variables are output, and then the LSTM model is applied for prediction. The three-dimensional port collaborative prediction scheme takes the historical monthly port cargo throughput of the three ports as the input variable of the forecasting model and uses the next month's cargo throughput of the target port as the output variable, and then applies the LSTM model for prediction.

In the second step, the three ports historical monthly cargo throughput data are collected and sorted as the original dataset, and the original dataset is divided into the training dataset and the test dataset in the ratio of 7.5 : 2.5.

In the third step, the training dataset is used to train the LSTM prediction model, and the experimental parameters are repeatedly modified to optimize the prediction accuracy of the LSTM model.

In the fourth step, the test training set is used as the input index, and the prediction results and errors of the 12 prediction schemes are obtained by the LSTM prediction model.

In the fifth step, the predicted values and errors of each prediction scheme are compared and analyzed.

### 3.3. Model Parameters and Performance Indicators

The prediction model proposed in this study was designed using MATLAB 2017a and Linux system environment using Python 2.7 programming. The LSTM model parameters are Epoch = 5000, Hidden_layer = 100, LR = 0.002, step = 2.

To accurately assess the effectiveness of the experimental model, three evaluation metrics, mean absolute percentage error (MAPE), root mean squared error (RMSE), and mean absolute error (MAE), are used in this paper to quantitatively evaluate the prediction performance of the proposed model. The smaller the value, the higher the accuracy of the model, and the better the prediction performance. Among them, MAPE is used as the main evaluation index. The formulas for these three evaluation criteria are shown below:

(7) $$\begin{array}{c} MAPE=\frac{1}{n}\sum_{i=1}^{n}\left|\frac{x_{g}-x_{p}}{x_{g}}\right|\times 100\%,\\ RMSE=\sqrt{\frac{1}{n}\sum_{i=1}^{n}{\left(x_{g}-x_{p}\right)}^{2}},\\ MAE=\frac{1}{n}\sum_{i=1}^{n}\left|x_{g}-x_{p}\right|, \end{array}$$

where *x*_*g*_ is the actual value and *x*_*p*_ is the corresponding predicted value.

## 4. Experiment

### 4.1. Data Description and Partition

The research object of this paper is the three ports around the Bohai Sea. The Bohai Rim region refers to the Bohai Rim Economic Region with the Beijing-Tianjin-Hebei as the core, and the Liaodong Peninsula and the Shandong Peninsula as the two wings. It is located in the northern part of China along the west coast of the Pacific Ocean, including Beijing, Tianjin, Tangshan, Qinhuangdao, Dalian, Yantai, Weihai, Qingdao, Dongying, Baoding, Shijiazhuang, Jinan, Shenyang, and many other cities. The Bohai Sea Port Group is one of China's five major port groups and plays an important role in China's open coastal development strategy.

**Figure 4:**
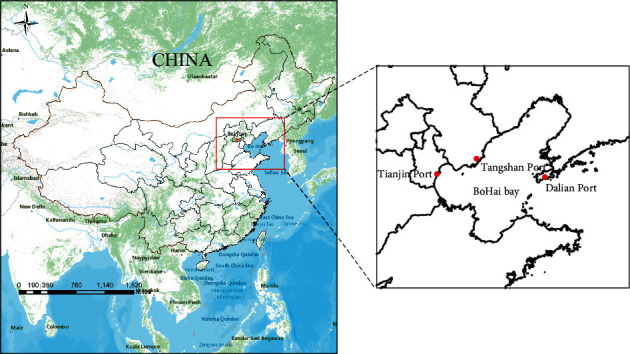
Geographical location of port.

Based on the geographical location characteristics of the ports around the Bohai Sea and the cargo throughput of each port, the three ports of Tianjin Port, Dalian Port, and Tangshan Port were selected as research objects. The geographical location of each port is shown in Figure 4.

**Figure 5:**
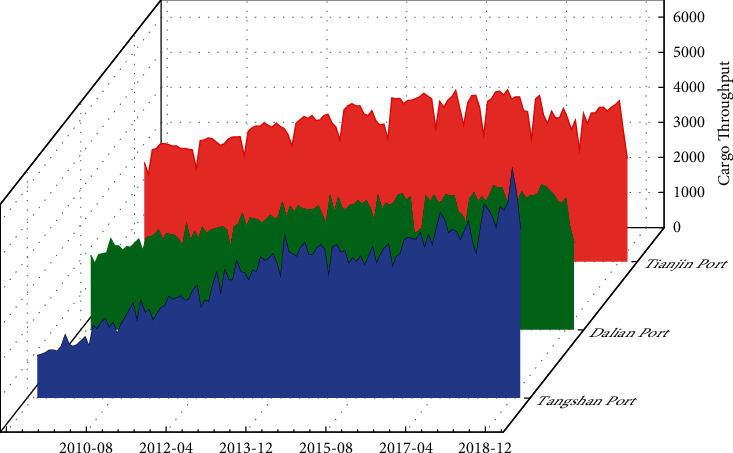
Monthly cargo throughput by port.

Tianjin Port is located at the mouth of the Haihe River in Tianjin, China, as an important area of the Bohai Economic Circle. Dalian Port is located in the Dalian Bay at the southern end of the Liaodong Peninsula. It is at the center of the Northeast Asian economic circle and is one of the most important comprehensive foreign trade ports in the Northeast. Tangshan Port is located at the north bank of the Bohai Bay in Tangshan City, Hebei Province. It is located in the center of the Bohai Sea and the Beijing-Tianjin Double Economic Circle, including the Jingtang Port Area and the Caofeidian Port Area. It is an important component of China's energy, raw materials, and other specialized materials transportation systems. The data used in this study is the monthly cargo throughput of the above three ports from March 2009 to January 2019, obtained from the live data released by the China Port Network. The raw data of the cargo throughput of each port is shown in Figure 5.

**Figure 6:**
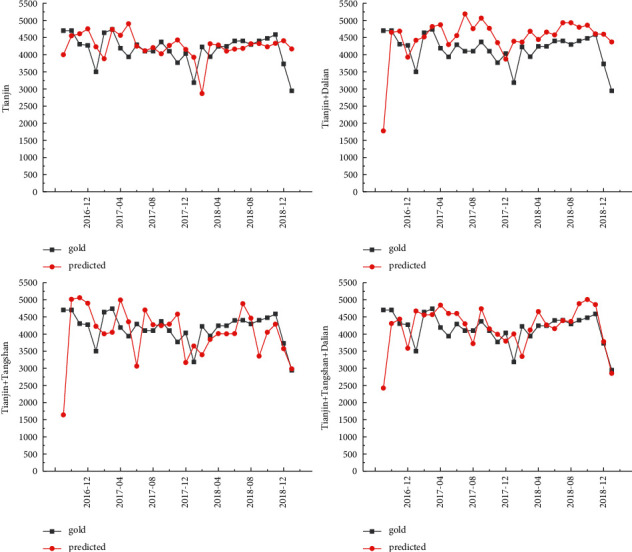
Forecast results of Tianjin port.

It can be seen from Figure 6 that the cargo throughput of each port generally shows an upward trend and there is a certain periodicity. In order to facilitate the model processing, 121 data of 3 ports are divided into two data sample sets: training sample and test sample. The data of the training sample set was selected from January 2009 to August 2016. A total of 92 data were collected. The data of the test samples were selected from September 2016 to January 2019.

### 4.2. Results

#### 4.2.1. Tianjin Port Cargo Throughput Forecast Results

The cargo throughput forecast of Tianjin Port is based on the design steps of the experimental method. The LSTM model is used to test the four prediction schemes of Tianjin Port, Tianjin Port + Dalian Port, Tianjin Port + Tangshan Port, and Tianjin Port + Dalian Port + Tangshan Port. MAPE was selected as its error evaluation index. The forecast results of Tianjin Port's cargo throughput are shown in Table 1 and Figure 6. The minimum error is 10.08%. The comparison further reveals that the Tianjin Port + Dalian Port + Tangshan Port forecasting scheme shows the least error in the coordinated forecasting scheme. Interestingly, it is noticed that when forecasting the cargo throughput of Tianjin Port, the cargo throughput indicators of Dalian Port and Tangshan Port are added to make the forecast results more accurate.

#### 4.2.2. Dalian Port Cargo Throughput Forecast Results

**Figure 7:**
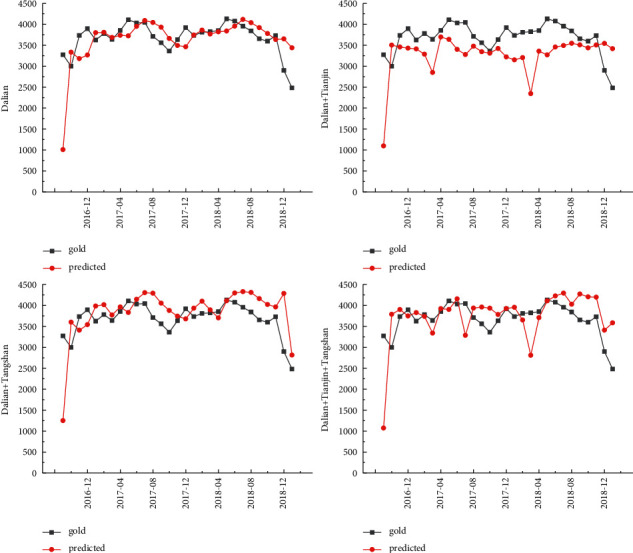
Forecast results of Dalian port.

The cargo throughput forecast of Tianjin Port is based on the design steps of the experimental method. The LSTM model is used to test the four prediction schemes of Dalian Port, Dalian Port + Tianjin Port, Dalian Port + Tangshan Port, and Dalian Port + Tianjin Port + Tangshan Port. MAPE was selected as error evaluation index. The cargo throughput forecast results of Dalian Port are shown in Table 2 and Figure 7. The minimum error is 9.65%. The comparative analysis further reveals that the Dalian Port + Tangshan Port prediction scheme has the smallest error in the coordinated prediction scheme. It can be seen that while predicting the cargo throughput of Dalian Port, the throughput index of joining Tangshan Port is the best, and the impact of Tangshan Port on the cargo throughput of Dalian Port is greater than that of Tianjin Port.

#### 4.2.3. Tangshan Port Cargo Throughput Forecast Results

**Figure 8:**
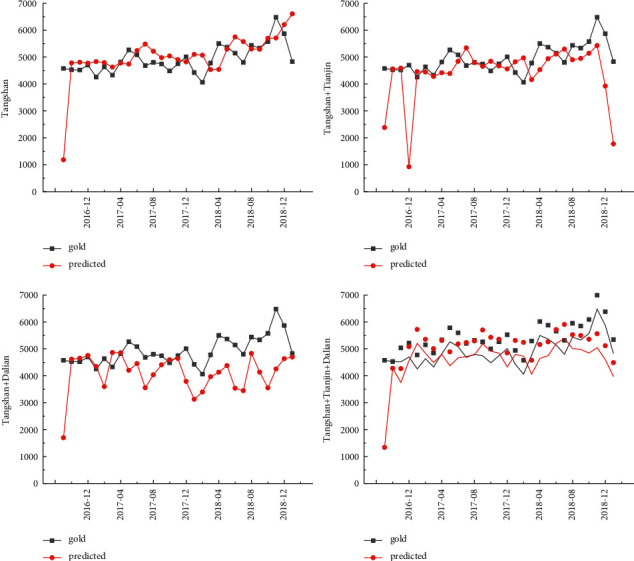
Forecast results of Tangshan port.

The cargo throughput forecast of Tianjin Port is based on the design steps of the experimental method. The LSTM model is used to test the four prediction schemes of Tangshan Port, Tangshan Port + Tianjin Port, Tangshan Port + Dalian Port, and Tangshan Port + Tianjin Port + Dalian Port. MAPE was selected as the error evaluation index. The cargo throughput forecast results of Tangshan Port are shown in Table 3 and Figure 8. The minimum error is 11.27%. Through comparison, it is found that the prediction scheme of Tangshan Port + Tianjin Port + Dalian Port is the smallest in the coordinated prediction scheme. It can be observed that when forecasting the cargo throughput of Tangshan Port, the cargo throughput indicators of Tianjin Port and Dalian Port are more accurate.

#### 4.2.4. Model Accuracy Comparison

Before entering the discussion section, in order to more fully evaluate the prediction performance of the LSTM model, which has reached the final model to achieve optimality, this paper compares the LSTM model with other models, including ARIMA, GBRT, and some popular deep learning models. To make the comparison results more obvious and concise, the prediction results of the best of the three ports are used to compare with other models. And MAPE is used as the comparison metric. With all other conditions held constant, the final results are shown in Table 4. From the results, it can be seen that the LSTM model proposed in this paper is more effective than the other prediction models.

From the comparison results, we can see that the prediction accuracy of the LSTM model is significantly better than other models. It may be because the LSTM model method is more advanced. It is a novel method in the field of artificial intelligence, with the ability to learn longer time series, which can mine effective feature representations from a large amount of input data. The interference of features such as volatility and noisiness of air pollutant data is avoided, and the prediction stability is improved.

### 4.3. Discussion

The historical cargo throughput of Tianjin Port, Dalian Port, and Tangshan Port has a certain periodicity, showing an overall upward trend, but the respective trends are obviously different. This is because although the three ports are in the Bohai Rim region, the economic hinterland of each port has both overlapping areas and areas covered by them. The economic hinterland of Tianjin Port and Tangshan Port is dominated by Beijing, Tianjin, North China,and Northwest China. Among them, the direct economic hinterland includes Tianjin, Beijing, Hebei, and Shanxi provinces, and the indirect economic hinterland extends to the provinces of Shaanxi, Gansu, Ningxia, Qinghai, Xinjiang, Inner Mongolia, Sichuan, Tibet, and Mongolia through the integrated transportation network area. The economic hinterland of Dalian Port is mainly Shandong Province, the three northeastern provinces, and Inner Mongolia. The differences in the coverage areas of the three ports and the different economic strengths of their respective hinterlands have affected the port's cargo throughput and its changing trend to some extent.

In the analysis of the results of the combined forecasting scheme, it is found that the cargo throughput of Tangshan Port has a strong predictive ability for the cargo throughput of Tianjin Port and Dalian Port. In the forecast of cargo throughput of Tianjin Port, the predicted result of the Tianjin Port + Dalian Port + Tangshan Port combination plan is 10.08%. On the other hand, forecasting of cargo throughput of Dalian Port, the predicted result of the Dalian Port + Tangshan Port combination plan is 11.45%. Whereas, forecasting cargo throughput of Tangshan Port, the predicted result of the Tangshan Port + Tianjin Port + Dalian Port combination scheme is 12.31%. Tangshan Port is the youngest port in China, and its location is between Tianjin Port and Dalian Port. In 2018, Tangshan Port ranked fourth in the global ports with a cargo throughput of 630 million tons, an increase of 11.14%, ranking first in the world's top ten ports. The sharp increase in cargo throughput of Tangshan Port had a non-negligible impact on the cargo throughput of Tianjin Port and Dalian Port.

There are mutual influences and differences between the ports. The forecasting accuracy of Tianjin Port + Tangshan Port forecasting scheme is better than Tianjin Port + Dalian Port forecasting scheme, which indicates that Tangshan Port has a greater impact on the forecast of Tianjin Port cargo throughput; Dalian Port + Tangshan Port forecasting scheme has better prediction accuracy than Dalian Port + Tianjin port forecasting scheme showing that Tangshan Port has a greater impact on the forecast of Dalian Port cargo throughput; the forecast accuracy of Tangshan Port + Tianjin Port Forecasting Plan is better than that of Tangshan Port + Dalian Port Forecasting Plan, which indicates that Tianjin Port forecast of cargo throughput of Tangshan Port is even greater. This is because the distance between Tangshan Port and Tianjin Port is shorter than that between Tangshan Port and Dalian Port, and most of the economic hinterland covered by Tianjin Port and Tangshan Port is repeated; hence, the cargo handling demand is similar. Moreover, the Tianjin Port Group and the Tangshan Port Group have synergistic cooperation and the integration of soft and hard resources for container transportation. Therefore, the mutual influence between Tangshan Port and Dalian Port is deeper.

The forecast of port cargo throughput is not only affected by historical data but also by the cargo throughput of nearby ports. Therefore, it is necessary to use historical data and data from nearby ports as input indicators. In order to obtain reliable prediction results, this study used a multilayer LSTM model, and after repeated experiments, the final prediction results were obtained. The results show that the LSTM model can solve effectively both the nonlinear and volatility problems, and has good predictive ability for port cargo throughput. It can provide reference for practical work, improve the overall efficiency of the target port, and reduce operating costs.

## 5. Conclusions

LSTM artificial neural method was used as port cargo throughput collaborative predive model for the three Bohai Rim ports. Twelve prediction schemes are designed to predict cargo throughput of Tianjin Port, Dalian Port, and Tangshan Port as the research objects. The research conclusions are as follows:

The MAPE of all the three port cargo throughput forecasts are below 18%, and the average MAPE of all the combined schemes is 11.76%, indicating that the LSTM model is suitable for port cargo throughput forecasting. The LSTM prediction model can accurately predict the port cargo throughput and can effectively solve the nonlinear time series prediction problem as compared to the general linear prediction model. Since the experimental data in this paper is based on three Bohai Sea ports data, the data obtained is however limited. If the data can be collected more comprehensively as a training sample, the predicted value of the model may be better. Therefore, the LSTM prediction model has strong applicability and high application value in predicting port cargo throughput.

The forecasting analysis revealed that all the three ports exert mutual influence on each other despite of any differences. The combined forecasting model significantly improved the forecasting accuracy of port cargo throughput, and the stability also has improved substantially. It is also noticed that the combined forecasting scheme predicts the trend of port cargo throughput more accurately than the traditional single forecasting scheme. Therefore, it is believed that multiple port collaborative predictions can make the forecast results of port cargo throughput better and more accurate. In the subsequent port throughput forecasting problem, historical data of ports with strong influence on the target port can be used as input indicators for joint predictions.

The current work on forecasting cargo throughput of ports in the Bohai Rim region of China based on the LSTM model needs to be further extended. First, it is necessary to further understand the influencing factors among ports in the Bohai Rim region. Secondly, there is still room for improvement of the LSTM model in terms of predicting port throughput in terms of improving the prediction accuracy, such as the use of attention mechanism and other methods can be considered in the future.

## Figures and Tables

**Table 1:** Forecast results of Tianjin port.

Target port	Combination scheme	Test-MAPE	Test-RMSE	Test-MAE
Tianjin	Tianjin	10.2226491	3.82	2.73
	Tianjin + Dalian	14.88993	5.24	4.68
	Tianjin + Tangshan	13.8169734	4.96	3.85
	Tianjin + Dalian + Tangshan	10.0764947	3.62	2.37

**Table 2:** Forecast results of Dalian port.

Target port	Combination scheme	Test-MAPE	Test-RMSE	Test-MAE
Dalian	Dalian	9.6531761	3.82	2.73
	Dalian + Tianjin	15.3061097	7.58	6.85
	Dalian + Tangshan	11.4498638	4.05	2.99
	Dalian + Tianjin + Tangshan	12.0852955	4.65	3.69

**Table 3:** Forecast results of Tangshan port.

Target port	Combination scheme	Test-MAPE	Test-RMSE	Test-MAE
Tangshan	Tangshan	11.276331	3.84	2.83
	Tangshan + Tianjin	14.5784657	4.28	3.19
	Tangshan + Dalian	17.5350388	6.98	5.94
	Tangshan + Tianjin + Dalian	12.3145167	4.64	3.54

**Table 4:** Model accuracy comparison.

Target port	Best combination scheme	LSTM	ARIMA	GBRT	ANN	RNN
Tianjin	Tianjin + Dalian + Tangshan	10.08	17.87	14.65	19.67	18.05
Dalian	Dalian	9.65	19.18	12.58	20.58	16.84
Tangshan	Tangshan	11.28	15.67	15.94	27.79	20.55

## Data Availability

Raw data used to support the results of this study are included in the article.

## References

[1] Wiegmans B., Witte P., Spit T. (2015). Characteristics of European inland ports: a statistical analysis of inland waterway port development in Dutch municipalities. *Transportation Research Part A: Policy and Practice*.

[2] Rendon-Sanchez J. F., de Menezes L. M. (2019). Structural combination of seasonal exponential smoothing forecasts applied to load forecasting. *European Journal of Operational Research*.

[3] Xu W., Peng H., Zeng X., Zhou F., Tian X., Peng X. (2019). Deep belief network-based AR model for nonlinear time series forecasting. *Applied Soft Computing*.

[4] Scherrer W., Deistler M. (2019). Vector autoregressive moving average models. *Handbook of Statistics*.

[5] Klepsch J., Klüppelberg C., Wei T. (2017). Prediction of functional ARMA processes with an application to traffic data. *Econometrics and Statistics*.

[6] Chen S.-H., Chen J.-N. (2010). Forecasting container throughputs at ports using genetic programming. *Expert Systems with Applications*.

[7] Javedani Sadaei H., Enayatifar R., Gadelha Guimarães F., Mahmud M., Zakarya A. (2016). Alzamil,Combining ARFIMA models and fuzzy time series for the forecast of long memory time series. *Neuro computing*.

[8] Freire P. K. d M. M., Santos C. A. G., Silva G. B. L. d (2019). Analysis of the use of discrete wavelet transforms coupled with ANN for short-term streamflow forecasting. *Applied Soft Computing*.

[9] Geng J., Li M.-W., Dong Z.-H., Liao Yu-S. (2015). Port throughput forecasting by MARS-RSVR with chaotic simulated annealing particle swarm optimization algorithm. *Neurocomputing*.

[10] Muzaffar S., Afshari A. (2019). Short-term load forecasts using LSTM networks. *Energy Procedia*.

[11] Merkuryeva G., Valberga A., Smirnov A. (2019). Demand forecasting in pharmaceutical supply chains: a case study. *Procedia Computer Science*.

[12] Candelieri A., Giordani I., Archetti F. (2019). Tuning hyperparameters of a SVM-based water demand forecasting system through parallel global optimization. *Computers & Operations Research*.

[13] Suh D. Y., Ryerson M. S. (2019). Forecast to grow: aviation demand forecasting in an era of demand uncertainty and optimism bias. *Transportation Research Part E: Logistics and Transportation Review*.

[14] Tanizaki T., Hoshino T., Shimmura T., Takenaka T. (2019). Demand forecasting in restaurants using machine learning and statistical analysis. *Procedia CIRP*.

[15] Plakandaras V., Papadimitriou T., Gogas P. (2019). Forecasting transportation demand for the U.S. market. *Transportation Research Part A: Policy and Practice*.

[16] Law R., Li G., Fong D. K. C., Han X (2019). Tourism demand forecasting: a deep learning approach. *Annals of Tourism Research*.

[17] Matyjaszek M., Riesgo Fernández P., Krzemień A., Wodarski K., Fidalgo Valverde G. (2019). Forecasting coking coal prices by means of ARIMA models and neural networks, considering the transgenic time series theory. *Resources Policy*.

[18] Gonzalez J., Yu W. (2018). Non-linear system modeling using LSTM neural networks. *IFAC-PapersOnLine*.

[19] Xu L., Zhou X., Tao Y. (2021). Intelligent security performance prediction for IoT-enabled healthcare networks using improved CNN. *IEEE Transactions on Industrial Informatics*.

[20] Zhao J., Mao X., Chen L. (2019). Speech emotion recognition using deep 1D & 2D CNN LSTM networks. *Biomedical Signal Processing and Control*.

[21] Zhou F., Hang R., Liu Q., Yuan X. (2019). Hyperspectral image classification using spectral-spatial LSTMs. *Neurocomputing*.

[22] Yu R., Gao J., Yu M. (2019). LSTM-EFG for wind power forecasting based on sequential correlation features. *Future Generation Computer Systems*.

[23] Yildirim O., Baloglu U. B., Tan Ru-S., Ciaccio E. J., Acharya U. R. (2019). A new approach for arrhythmia classification using deep coded features and LSTM networks. *Computer Methods and Programs in Biomedicine*.

[24] Makarenkov V., Rokach L., Shapira B. (2019). Choosing the right word: using bidirectional LSTM tagger for writing support systems. *Engineering Applications of Artificial Intelligence*.

[25] Majd M., Safabakhsh R. (2019). Correlational Convolutional LSTM for Human Action Recognition. *Neurocomputing*.

[26] Kim C. B. (2016). Impact of exchange rate movements, global economic activity, and the BDI volatility on loaded port cargo throughput in South Korea. *The Asian Journal of Shipping and Logistics*.

[27] Yang B., Sun S., Li J., Lin X., Tian Y. (2019). Traffic flow prediction using LSTM with feature enhancement. *Neurocomputing*.

[28] Qin Y., Song D., Chen H. (2017). A Dual-Stage Attention-Based Recurrent Neural Network for Time Series Prediction. *Time Series Prediction*.

[29] Zheng W., Zhao P., Huang K. Understanding the property of long term memory for the LSTM with attention mechanism.

[30] Johannesen N. J., Kolhe M., Goodwin M. (2019). Relative evaluation of regression tools for urban area electrical energy demand forecasting. *Journal of Cleaner Production*.

[31] Cheikhrouhou N., Marmier F., Ayadi O., Wieser P. (2011). A collaborative demand forecasting process with event-based fuzzy judgements. *Computers & Industrial Engineering*.

[32] Chen T. (2013). A collaborative and artificial intelligence approach for semiconductor cost forecasting. *Computers & Industrial Engineering*.

[33] Gao L. (2015). Collaborative forecasting, inventory hedging and contract coordination in dynamic supply risk management. *European Journal of Operational Research*.

[34] Wang Yi-C., Toly Chen T. C (2018). A direct-solution fuzzy collaborative intelligence approach for yield forecasting in semiconductor manufacturing. *Procedia Manufacturing*.

[35] Thomson M. E., Pollock A. C., Önkal D., Gönül M. S. (2019). Combining forecasts: performance and coherence. *International Journal of Forecasting*.

[36] Chen T. (2015). Analyzing and forecasting the global CO2 concentration - a collaborative fuzzy-neural agent network approach. *Journal of Applied Research and Technology*.

[37] Rashed Y., Meersman H., Sys C., Van de Voorde E., Vanelslander T. (2018). A combined approach to forecast container throughput demand: scenarios for the Hamburg-Le Havre range of ports. *Transportation Research Part A: Policy and Practice*.

[38] Gosasang V., Chandraprakaikul W., Kiattisin S. (2011). A comparison of traditional and neural networks forecasting techniques for container throughput at Bangkok port. *The Asian Journal of Shipping and Logistics*.

[39] Xie G., Zhang N., Wang S. (2017). Data characteristic analysis and model selection for container throughput forecasting within a decomposition-ensemble methodology. *Transportation Research Part E: Logistics and Transportation Review*.

[40] Mo L., Xie L., Jiang X., Teng G., Xu L., Xiao J. (2018). GMDH-based hybrid model for container throughput forecasting: selective combination forecasting in nonlinear subseries. *Applied Soft Computing*.

[41] Niu M., Hu Y., Sun S., Liu Yu (2018). A novel hybrid decomposition-ensemble model based on VMD and HGWO for container throughput forecasting. *Applied Mathematical Modelling*.

[42] Li H., Bai J., Li Y. (2019). A novel secondary decomposition learning paradigm with kernel extreme learning machine for multi-step forecasting of container throughput. *Physica A: Statistical Mechanics and Its Applications*.

[43] Du P., Wang J., Yang W., Tong N. (2019). Container throughput forecasting using a novel hybrid learning method with error correction strategy. *Knowledge-Based Systems*.

